# Frequency of satisfaction and dissatisfaction with practice among rural-based, group-employed physicians and non-physician practitioners

**DOI:** 10.1186/s12913-016-1777-8

**Published:** 2016-10-22

**Authors:** Anthony C. Waddimba, Melissa Scribani, Nicole Krupa, John J. May, Paul Jenkins

**Affiliations:** 1 Bassett Healthcare Network, Research Institute, 1 Atwell Road, Cooperstown, NY 13326 USA; 2 Columbia University College of Physicians and Surgeons, 630 West 168th St, New York, NY 10032 USA; 3 Columbia University Mailman School of Public Health, 722 West 168th St, New York, NY 10032 USA

**Keywords:** Job satisfaction, Quality of work life, Rural healthcare, Health workforce, Physicians, Nurse practitioners, Physician assistants, Inflated beta regression

## Abstract

**Background:**

Widespread dissatisfaction among United States (U.S.) clinicians could endanger ongoing reforms. Practitioners in rural/underserved areas withstand stressors that are unique to or accentuated in those settings. Medical professionals employed by integrating delivery systems are often distressed by the cacophony of organizational change(s) that such consolidation portends. We investigated the factors associated with dis/satisfaction with rural practice among doctors/non-physician practitioners employed by an integrated healthcare delivery network serving 9 counties of upstate New York, during a time of organizational transition.

**Methods:**

We linked administrative data about practice units with cross-sectional data from a self-administered multi-dimensional questionnaire that contained practitioner demographics plus valid scales assessing autonomy/relatedness needs, risk aversion, tolerance for uncertainty/ambiguity, meaningfulness of patient care, and workload. We targeted medical professionals on the institutional payroll for inclusion. We excluded those who retired, resigned or were fired during the study launch, plus members of the advisory board and research team. Fixed-effects beta regressions were performed to test univariate associations between each factor and the percent of time a provider was dis/satisfied. Factors that manifested significant fixed effects were entered into multivariate, inflated beta regression models of the proportion of time that practitioners were dis/satisfied, incorporating clustering by practice unit as a random effect.

**Results:**

Of the 473 eligible participants. 308 (65.1 %) completed the questionnaire. 59.1 % of respondents were doctoral-level; 40.9 % mid-level practitioners. Practitioners with heavier workloads and/or greater uncertainty intolerance were less likely to enjoy top-quintile satisfaction; those deriving greater meaning from practice were more likely. Higher meaningfulness and gratified relational needs increased one’s likelihood of being in the lowest quintile of dissatisfaction; heavier workload and greater intolerance of uncertainty reduced that likelihood. Practitioner demographics and most practice unit characteristics did not manifest any independent effect.

**Conclusions:**

Mutable factors, such as workload, work meaningfulness, relational needs, uncertainty/ambiguity tolerance, and risk-taking attitudes displayed the strongest association with practitioner satisfaction/dissatisfaction, independent of demographics and practice unit characteristics. Organizational efforts should be dedicated to a redesign of group-employment models, including more equitable division of clinical labor, building supportive peer networks, and uncertainty/risk tolerance coaching, to improve the quality of work life among rural practitioners.

## Background

The widespread dissatisfaction among United States (U.S.) clinicians could endanger the success of ongoing systemic reforms [1]. Rapid change in the healthcare landscape is exerting enormous psychological pressure on practitioners by escalating regulatory/documentation requirements, increasing risk of malpractice litigation, imposing value-based purchasing or accountable-care standards, and eroding clinical autonomy [2–6]. The new focus on patient-centered medicine has often relegated practitioner well-being to the back seat [7]. Yet numerous reports highlight the increasing discontent, distress, frustration, and burnout among medical professionals [8–11]. U.S. physicians are more prone to burnout [12], and more likely to engage in suicide ideation or to die from work-related suicide than other working adults [13, 14]. It sometimes seems questionable who are the more distressed group, practitioners or patients [15]. Practitioner dissatisfaction is linked to patient dissatisfaction [16], inferior prescribing [17], suboptimal care [18], and surgery errors [19]. It foments professional withdrawal behaviors such as change of specialty, change of organization, unwillingness to mentor trainees, not recommending a medical career, and/or leaving medicine [20, 21], which threaten long-term stability of the clinician workforce. Loss of a practitioner disrupts continuity of care for patients and forces other practitioners to take on a higher workload in the interim [22]. The total cost of replacing a departing fulltime physician ranges from several hundred thousand to over one million dollars [23, 24], making practitioner dissatisfaction very expensive for healthcare organizations.

Rural practitioners, unlike their urban counterparts, face unique challenges such as lengthy distances between service access points, geographical or cultural isolation, resource-poor under-insured and underserved patient populations, and lower rates of reimbursement/remuneration [25–27]. Dual relationships such as having a patient who doubles as a neighbor or friend or minister, are common in close-knit rural communities [28]. The work life of rural-based practitioners thus includes the additional stressor of balancing hard-to-navigate private and professional boundaries [29–31]. Yet practitioners of a certain disposition [32] might prefer such familiarity and community bonds [33, 34] to the anonymity of urban practice. Data on work-related wellbeing among rural practitioners, during the contemporary wave of systemic and institutional reforms, is still quite fragmented [35].

Given the competition in the current healthcare marketplace, many hospitals are merging with physician groups to form accountable-care organizations (ACOs) and most hitherto autonomous solo or group practices will likely face mergers or outright purchase by larger hospitals, resulting in more integrated delivery systems [36, 37]. Organizational restructuring in an integrating and consolidating healthcare delivery system can be seen by medical practitioners as a threat to their professional identity and workplace wellbeing [38]. An increasing proportion of medical professionals will become salaried employees in these integrating systems [39]. Practitioners who work as group-model employees face further constraints on their clinical autonomy and caps on their earning potential. Some reports suggest that employed practitioners are more dissatisfied than their independent counterparts [40]. In one study, hospital-employed family practitioners were less satisfied with being a physician than independent counterparts, and were more likely to leave practice [41]. Further research on the factors that exert the strongest influence on job dis/satisfaction among medical practitioners who are employed in integrated systems can inform efforts that seek to optimize wellbeing by balancing institutional goals with practitioners’ professional needs during and after consolidation [42].

Studies of job satisfaction among rural practitioners are often limited to one section of the workforce, e.g. physicians [43], physician assistants [44], or nurse practitioners [45]. Yet medical professionals from diverse disciplines increasingly work together on the same team [46]. We triangulated information from self-administered surveys with administrative data at the service unit level in a study whose objective was to investigate factors associated with the frequency of satisfaction and dissatisfaction with practice among physician, pharmacist, nurse practitioner, and physician assistant employees of a healthcare network serving nine rural counties of upstate New York. We aimed to compare factors that predict satisfaction but not dissatisfaction, dissatisfaction but not satisfaction, or both satisfaction and dissatisfaction. Unlike prior studies, we utilized a measure of affective job satisfaction based on fluctuation in the valence of job-related affect, and assessing the frequency of feeling satisfied or dissatisfied. Our purpose was to inform efforts at minimizing practitioner distress during changes to more integrated delivery systems in underserved/rural settings.

### Conceptual model

Job satisfaction was defined in this study as “a pleasurable or positive emotional state resulting from the appraisal of one’s job or job experiences” [47]. We focused on *global* job satisfaction, i.e. overall feelings about one’s practice as a whole, and not on *facet* satisfaction, i.e. feelings about specific aspects of one’s practice [48]. This definition highlights the affective component of job satisfaction [49]. Streams of workplace events do generate positive or negative emotional reactions and mood changes among individuals [50], which influence their happiness at work. Affective reactions, unlike personal traits, tend to fluctuate from day to day and setting to setting [49, 51]. In our conceptualization, the frequency (more than the intensity) of a practitioner’s happiness or unhappiness [52] with their practice determines their overall satisfaction or dissatisfaction.

**Fig. 1:**
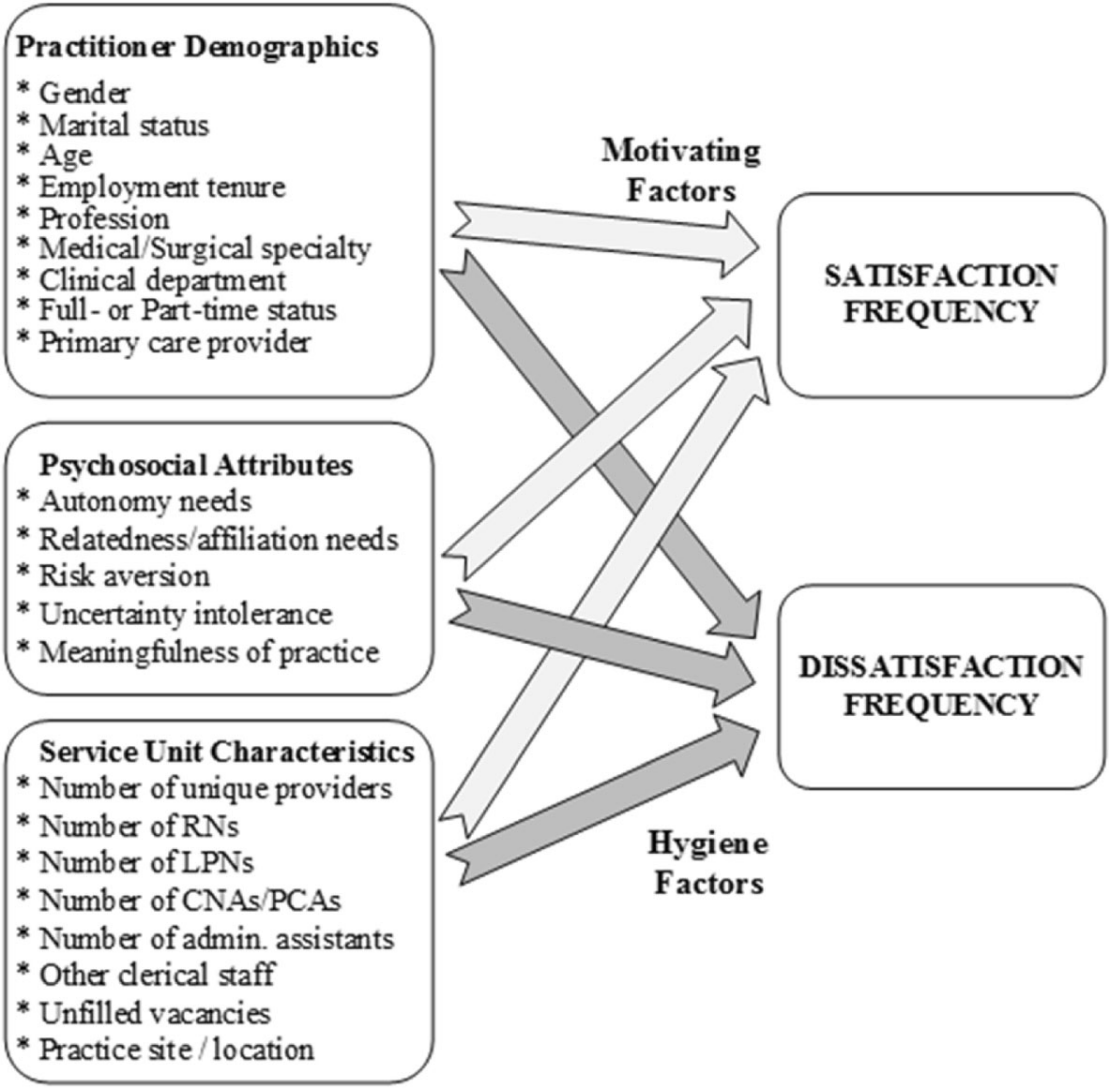
Conceptual model of satisfaction and dissatisfaction with practice among rural practitioners

The Motivation-Hygiene theory posits that some factors (*motivators*) influence job satisfaction but not dissatisfaction, and others (*hygiene factors*) cause dissatisfaction when absent/inadequate but hardly affect satisfaction [53]. We investigated factors that are motivators (affect satisfaction, not dissatisfaction), hygiene factors (predict dissatisfaction alone), and both (predict satisfaction and dissatisfaction). We further treated job dis/satisfaction as a function of the degree to which a practitioner’s professional needs are fulfilled or unfulfilled by their practice, i.e. concordance between a practitioner’s needs and the need-gratifying capacity of their clinical work [54]. Self-determination Theory (SDT) posits that goal-directed behavior is motivated by the drive to satisfy three universal innate needs: autonomy, relatedness, and competence [55]. We focus, in this study, on personal needs for autonomy and relatedness as predictors of job satisfaction. We incorporate additional dimensions that link workplace well-being to: perceived meaningfulness of work [56, 57], risk aversion [58] (e.g. due to fear of malpractice lawsuits [5]), intolerance of ambiguity or uncertainty [59], work load [60], and job demands [61]. We finally include contextual factors such as practitioners’ personal attributes and work environment characteristics in our conceptual model. See Fig. 1 for an illustration of this model.

## Methods

### Study design

This study analyzes cross-sectional observational data generated as part of the *Practitioner Resilience, Adaptability and Well-Being Study*, a longitudinal research project investigating a community of practitioners serving in central New York [62, 63].

### Study setting and context

The study was conducted within the context of a rural integrated health services delivery system with a catchment population dispersed over a nine-county region of central New York covering 5,600 square miles. This network comprises a 180-bed Columbia University-affiliated academic medical center, six community hospitals, 31 outreach clinics/primary practices, a long term care facility and 20 school-based health centers. It has enjoyed a majority share of the market for health services provision to large portions of this catchment population, with scarce competition from other payers, since the 1930’s. The network receives approximately 700,000 ambulatory care visits annually.

The medical staff each entered an individual contract with the organization to be part of a multispecialty group-employed practice under a capitated reimbursement model. The study occurred against the backdrop of significant organizational changes such as the rolling out of a network-wide EPIC™-based electronic medical record, impending retirement of the long-time central executive officer, management pressure on clinicians to increase the volume of patients seen, and budget deficit-reduction measures such as a hiring freeze on non-essential staff. This was in addition to changes in clinical practice resulting from the escalating state and federal mandates attached to health system reforms, and pre-existing stressors inherent in the institution’s isolated, rural setting.

### Sampling and data collection

#### Sample selection

In order to be considered for inclusion in the study, an individual had to be a salaried clinical staff member on the institutional payroll. We excluded temporary staff on *locum tenens* from the study owing to their lack of binding ties to the institution. We further excluded residents/trainees. Among prospective recruits (*N* = 493), 5 resigned or were terminated during the final planning and promotion phase of the study, 3 left clinical practice altogether, and 12 served on the study team or its advisory board and were excluded, leaving a study sample of 473 clinicians.

#### Survey/Questionnaire

The main data collection instrument was a 5-page self-administered questionnaire that combined demographic information and pre-validated, reliable scales measuring psychological needs, risk profile, meaningfulness, burnout, resilience, and job satisfaction (measures described below). The questionnaire was tested and piloted among members of the Advisory Board and Internal Medicine residents. The final version took an average of 15–20 min to complete. Its distribution was preceded by wide publicity at Medical Staff and department meetings with the help of opinion leaders and local champions. The rollout strategy followed standard procedures in the literature [64, 65]. A letter announcing the survey was e-mailed by the Research Institute Director to the Medical Staff listserv, followed 5 days later by an e-mail from the Advisory Board. Questionnaires were distributed both as a SurveyMonkey® hyperlink by e-mail and a hard-copy form via the network’s inter-office mailing system. Reminders were e-mailed to nonresponders at two and four weeks from the start date. Those still unresponsive after six weeks received a repeat solicitation with the e-mail hyperlink and hard-copy questionnaire. Aided by local champions, we conducted further publicity at various division/departmental meetings with a final round of surveys sent electronically and by hardcopy to nonresponders at the 12-week point.

#### Administrative data

Supplementary information, at the aggregate level of the service unit and clinical department, was obtained from the institution and merged with the survey data.

### Measures and variables

Principal Outcome(s): Self-reported satisfaction with practice was the main outcome for this study. This was captured by a single questionnaire item that asked respondents to estimate a percentage of time that they were satisfied and a percentage of time that they were not satisfied with practice. The item is an adaptation of the positive affect frequency estimates in the Fordyce Emotions Questionnaire [66], which is under-utilized even though its internal consistency reliability compares favorably with other measures of subjective well-being [51, 67]. This Fordyce item has been used to assess happiness with work in other studies [68] but we found no reports in the literature of its adaptation to job satisfaction among medical practitioners. Global job satisfaction is conceptually unidimensional and is reliably captured by a single questionnaire item [69].

Needs: We utilized the *autonomy* and *relatedness* subscales of the Basic Psychological Needs at Work Scale [70] to assess gratification of needs for autonomy and relatedness. The autonomy subscale comprises four items (e.g. “I can use my judgment when solving work-related problems”) and the relatedness subscale consists of four items (e.g. “When I’m with the people from my work environment, I feel understood”). All items are positively worded and use a six-point Likert-style response format ranging from 1 (strongly disagree) to 6 (strongly agree). Each subscale is scored by deriving the mean of its constituent items.

Work Meaningfulness: was assessed by the *Personal Meaning in Patient Care* scale [71], in which respondents rate the extent to which they derive “a sense of personal meaning” in their work with patients. The scale comprises six items (e.g. “Feeling deep connections with my patients”), each with a four-point Likert-style response format ranging from 1 (not at all) to 4 (a great deal), and is scored by summing up all the items.

Risk aversion: was captured by two items from the six-item *Risk-Taking Scale* [58] of the Jackson Personality Inventory [72]. The two items selected were “I try to avoid situations that have uncertain outcomes” and “I rarely, if ever, take risks when there is another alternative”. Responses are on a four-point Likert-style scale ranging from 1 (strongly agree) to 4 (strongly disagree). They were reverse-coded and summated so that higher scores would indicate greater aversion to risk.

Tolerance of uncertainty/ambiguity: was assessed by two items from the 13-item Stress from Uncertainty subscale of the *Physicians’ Reactions to Uncertainty in Patient Care* Scale [59]. The items selected were: “The uncertainty of patient care often troubles me” and “I usually feel anxious when I’m not sure of a diagnosis”. We formatted responses on a four-point Likert-style scale ranging from 1 (strongly agree) to 4 (strongly disagree), which we reverse-coded and summed so that higher scores would indicate greater discomfort with uncertainty.

Work load: was captured by the *perceived workload scale,* developed specifically for the parent project and described in a previous report [62]. It consists of five items (e.g. “I feel stressed out from caring for too many patients”), each scored according to a four-point Likert-style frequency rating ranging from 1 (“Never [0 % of the time]”) to 4 (“Frequently [>75 % of the time]”). The “*perceived workload*” score is derived by summing up the five items, with a higher score indicating a heavier workload.

Practitioner demographics: included gender, marital status, fulltime status, age (years), profession, medical specialty, clinical department, scope of practice, direct patient care versus ancillary care, primary versus specialty/subspecialty care, and employment tenure with the organization (in years).

Practice unit characteristics: that were examined included geographic location, total number of practitioners on the unit, both total and per-practitioner numbers of administrative managers, nursing staff, other support staff, as well as unfilled vacancies or open positions.

### Statistical analysis

Both of the dependent variables were percentages. 25 % of values were between 80–100 % for percent of time satisfied and 50 % of values lay between 0 and 20 % for percent of time dissatisfied (see Fig. 1). Our analytic strategy investigated factors that predicted the likelihood of belonging to the top quintile in satisfaction (satisfied 80–100 % of the time) separately from being satisfied < 80 % of the time; and that of being within the bottom quintile in dissatisfaction (dissatisfied 0–20 % of the time) separately from being dissatisfied > 20 % of the time. For the purposes of statistical modeling, we reformatted the outcomes as proportions (with bounded values between 0 and 1). We then modeled the outcomes via a maximum likelihood generalized additive model for location, scale and shape (GAMLSS) with an inflated beta distribution. Beta distributions can take a wide variety of shapes and do not require the symmetry assumption necessary for ordinary least squares regression, so they are more flexible in modeling outcomes that are formatted as rates, proportions or percentages [73–77]. We thus assumed a beta distribution, rather than a normal distribution, for values of proportion of time satisfied that were less than 0.8 and of proportion of time dissatisfied that were above 0.2, with probability mass parameters for satisfaction frequencies of 0.8–1.0 and dissatisfaction frequencies between 0.0–0.2. In χ_0_-inflated models (χ_0_ being a value between 0 and 1), where the distribution of the inflation presents ≥ 2 credible partitions, modeling a wider interval (e.g. 0–0.2 or 0.8–1), rather than a probability anchored on a single value (e.g. 0 or 1), is justified [78]. The variance is not treated as a nuisance parameter [79] in an *inflated beta regression* model, but is explicitly modeled concurrently with the mean and inflation. Three generalized linear sub-models were fit using maximum likelihood estimation. The three functions modeled: (a) the mean of the beta distribution with a logit link (mean or location submodel); (b) the variance of the beta distribution with a log link (variance submodel); and (c) the probability mass parameter with a logit link (inflation submodel).^1^ An inflated beta regression fits three submodels because some factors can influence the mean without affecting variance or inflation parameters; or influence the variance with no effect on the mean or inflation; or influence the inflation but neither the mean nor variance. We tested associations between each explanatory variable and changes in the mean, variance, and inflation of the outcome(s) simultaneously. These univariate inflated beta regression models, whose purpose was variable selection, only included fixed effects. We used the standard criterion of *p* < 0.05 to determine significant mean, variance, and inflation regressors during the variable selection process.

Covariates that were significant in univariate mean, variance, or inflation sub-models were entered into an multivariable inflated beta regression [80, 81] model that incorporated the multivariate mean, variance and inflation associations as fixed effects and clustering by service unit as a random effect. Multivariable models were refined by dropping the mean, variance or inflation regressors that did not meet the significance threshold (*p* < 0.05). Variables were further assessed for removal by comparing goodness of fit, based on the Akaike Information Criterion (AIC) and Bayesian Information Criterion (BIC), between models with and without the variable. We synthesized final, parsimonious models incorporating only variables that manifested a significant multivariate association with change in the mean, variance, or inflation of the outcomes(s), after adjusting for other covariates and for clustering. All analyses were performed via Statistical Analysis Software (SAS) version 9.3 from SAS Inc. (Cary, NC). The NLMIXED procedure was used to implement univariate and multivariable zero- and one-inflated beta regression models. We excluded one extreme outlier for percent of time satisfied (0 %) from models for that outcome.

## Results

**Fig. 2:**
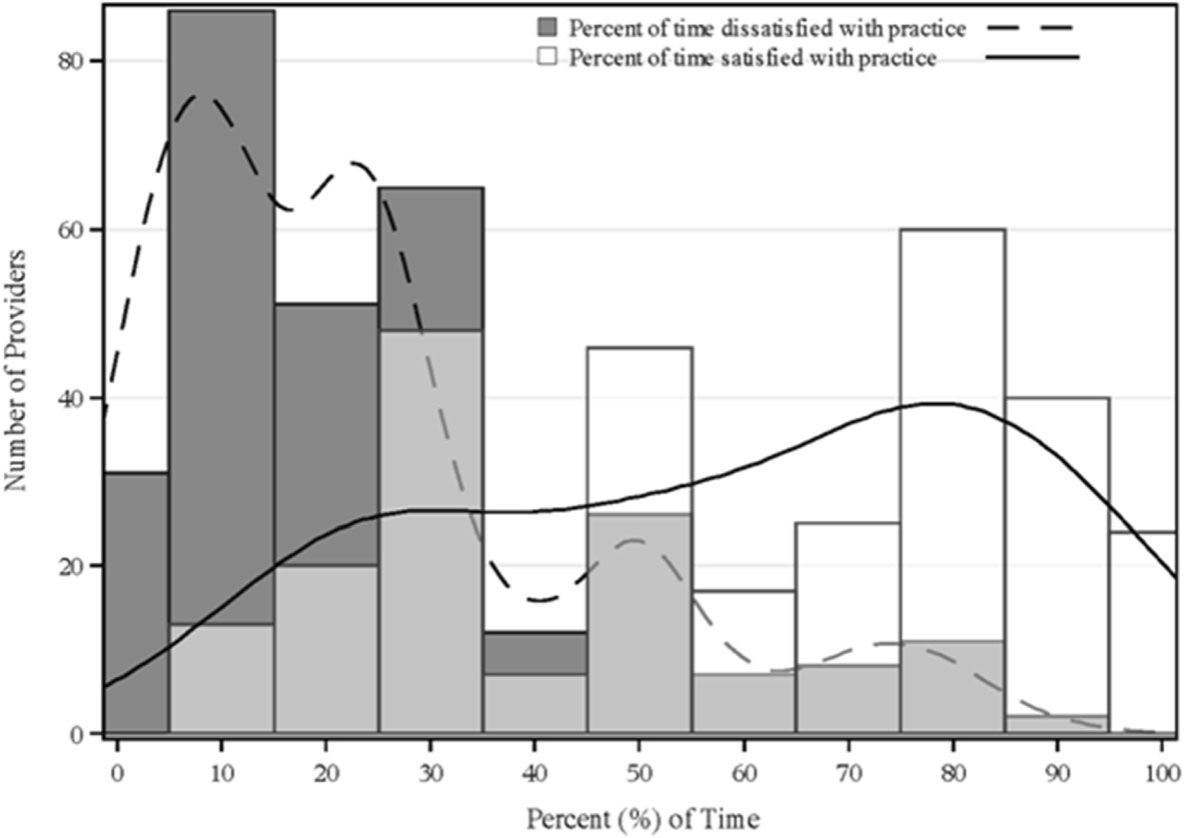
Distribution of satisfaction and dissatisfaction frequency among practitioners

308 (65.1 %) of the 473 survey recipients returned completed questionnaires. Respondents were 53.9 % male, 80.5 % married, 81.9 % fulltime employees, 59.1 % with doctoral degrees and 40.9 % advanced-practice clinicians. Their mean (95 % confidence interval) age and organizational tenure (95 % CI) were 49.2 (47.9, 50.6) and 10.3 (9.3, 11.3) years, respectively. 97.7 % of the respondents (*n* = 301) reported satisfaction ratings, and 97.4 % (*n* = 300) provided dissatisfaction ratings. The median (q1, q3) percent of time that practitioners were satisfied with their clinical practice was 60 (30, 80) %. They reported being dissatisfied at a median (q1, q3) frequency of 20 (10, 30) %. 30.6 % of the study sample were in the top quintile of satisfaction (satisfied ≥ 80 % of the time), whereas 54.7 % were in the bottom quintile in dissatisfaction (dissatisfied ≤ 20 % of the time). Figure 2 illustrates the distribution of professional satisfaction and dissatisfaction among the survey respondents. Table 1 depicts the characteristics of survey respondents.

**Table 1:** Characteristics of the survey respondents by satisfaction and dissatisfaction frequency

Variable	Total sample, *N* = 308	Satisfied ≥ 80 % of the Time, *N* = 92	Dissatisfied ≤ 20 % of the Time, *N* = 164
	*N* (%)	*N* (%)	*N* (%)
Male Gender	166 (53.9)	52 (56.5)	90 (54.9)
Married	248 (80.5)	80 (87.0)	141 (86.0)
Age < 45 years	117 (38.4)	36 (40.0)	64 (39.8)
Organizational Tenure < 15 years	228 (74.0)	69 (75.0)	119 (72.6)
Works Full Time	252 (81.8)	78 (84.8)	134 (81.7)
Works Part Time or Per Diem	56 (18.2)	14 (15.2)	30 (18.3)
Advanced-Practice Clinician/Non-Physician	126 (40.9)	40 (43.5)	73 (44.5)
Doctor	182 (59.1)	52 (56.5)	91 (55.5)
Primary Care Practitioner	99 (32.1)	21 (22.8)	43 (26.2)
Non-Primary Care Practitioner	209 (67.9)	71 (77.2)	121 (73.8)
Small Clinical Unit (Number of Practitioners ≤ 5)	92 (30.7)	25 (28.4)	43 (27.4)
	Median (Q_1_, Q_3_)	Median (Q_1_, Q_3_)	Median (Q_1_, Q_3_)
Number of Support Staff Full-time Equivalents (FTEs)	5.1 (2, 9)	4 (2, 7.6)	4 (2, 7.6)
Perceived Workload	12 (10, 14)	9 (8,12)	10 (8, 12)
Autonomy Needs	5.3 (4.8, 5.8)	5.8 (5, 6)	5.5 (5, 6)
Relatedness Needs	5.0 (4.3, 5.3)	5 (4.5, 5.8)	5 (4.8, 5.5)
Meaningfulness of Practice	3.3 (2.8, 3.7)	3.5 (3, 3,9)	3.4 (3.0, 3,8)
Risk Aversion	5 (4, 6)	5 (4, 6)	5 (4, 6)
Intolerance of Uncertainty/Ambiguity	5 (4, 6)	6 (4, 6)	5 (4, 6)
Percent of Time Satisfied	60 (30, 80)	90 (80, 95)	80 (70, 90)
Percent of Time Dissatisfied	20 (10, 30)	5 (2, 10)	10 (5, 15)

Table 2 outlines the results of the univariate fixed-effects beta regressions of the job satisfaction outcome on each explanatory variable. These variables had a significant, univariate association with changes in the mean of the beta distribution for the proportion of time that practitioners were satisfied: gender (male versus female), marital status (married versus unmarried), length of organizational tenure (≥ 15 versus < 15 years), fulltime (fulltime versus part-time or per diem) status, working in primary healthcare, perceived workload, autonomy needs, relational needs, and the perceived meaningfulness of practice. Those with a significant, unadjusted association with changes in the variance of the beta distribution for the frequency of satisfaction with practice were: gender, marital status, tenure, fulltime status, profession (doctor versus APC), and primary care specialty. A significant, unadjusted association with the likelihood of a practitioner feeling satisfied 80–100 % of the time was found with: being married, age group (< 45 versus ≥ 45 years), working fulltime, being an APC, working outside of primary care, perceived workload, autonomy needs, relational needs, work meaningfulness, tolerance of ambiguity and the levels of support staff on a unit.

**Table 2:** Univariate beta regressions^a^ of the proportion of time that practitioners were satisfied

Parameter	Mean sub-model	Variance sub-model	One-inflation sub-model
	Estimate (standard error)	Estimate (standard error)	Estimate (standard error)
Male gender	**0.3810 (.0573)**	**0.8669 (.0936)**	0.1495 (.1287)
Married	**0.4103 (.0601)**	**0.8296 (.0930)**	**0.3584 (.1412)**
Age < 45 years	−0.3709 (.3607)	−0.9584 (.9811)	**−1.8971 (.8408)**
Organizational Tenure < 15 years	**0.3775 (.0605)**	**0.9332 (.0911)**	0.2006 (.1274)
Works Fulltime	**0.2949 (.0628)**	**0.8542 (.0956)**	**0.2996 (.1380)**
Works Part-time/Per Diem	**0.5157 (.0793)**	**0.9182 (.1410)**	0.0170 (.2120)
Doctor	0.0014 (.0626)	**0.3838 (.1005)**	−0.2160 (.1368)
Advanced-Practice Clinician (APC)	−0.0815 (.0565)	**0.4197 (.0899)**	**−0.4034 (.1262)**
Works in Primary Care	**0.3846 (.0624)**	**0.8888 (.1061)**	−0.3182 (.1715)
Not in Primary Care	**0.3470 (.0566)**	**0.8457 (.0900)**	**0.3478 (.1280)**
Workload ψ	**−0.3111 (.0616)**	−0.1526 (.1183)	**−1.1312 (.1769)**
Autonomy Needs ψ	**0.2996 (.0599)**	−0.0582 (.0995)	**0.3714 (.1543)**
Relatedness Needs ψ	**0.3417 (.0609)**	−0.0958 (.1131)	**0.3697 (.1474)**
Meaningfulness of Practice ψ	**0.1877 (.0550)**	−0.0458 (.0885)	**0.4522 (.1455)**
Risk Aversion ψ	−0.0309 (.0548)	0.1397 (.0911)	−0.1849 (.1274)
Intolerance of Uncertainty ψ	−0.0805 (.0608)	0.0745 (.1000)	**−0.4309 (.1307)**
Unit Support Staff F.T.E.s ψ	0.0571 (.0473)	0.0755 (.0800)	**−0.4239 (.1625)**
Clinical Unit of ≤ 5 Practitioners	−0.3398 (.5085)	0.3983 (.6372)	−0.9243 (.7456)

Note: Bolded figures indicate statistical significance at the alpha = 0.05 significance level

Ψ = This continuous variable was standardized

^a^ These are fixed-effects-only one-inflated beta regressions, on each independent variable, of the proportion of time that a provider was satisfied with their clinical practice

Table 3 outlines the results of the univariate fixed-effects beta regressions of the dissatisfaction outcome on each covariate. These covariates had a significant, univariate association with the mean for the proportion of time that practitioners were dissatisfied: gender, marital status, fulltime status, being an APC, working in primary care, workload, autonomy needs, relational needs, and the size of a clinical unit. The significant variance regressors, in unadjusted models, were: gender, marital status, age group, fulltime status, profession, working in primary care or not, autonomy, risk aversion, and the size of a clinical unit. Significant regressors in unadjusted fixed-effects zero-inflation beta regression models were: gender, being married, age group, tenure category, fulltime status, being an APC, not working in primary care, workload, autonomy, relatedness, meaningfulness, risk aversion and tolerance of uncertainty.

**Table 3:** Univariate beta regressions^a^ of the proportion of time that practitioners were dissatisfied

Parameter	Mean sub-model	Variance sub-model	Zero-inflation sub-model
	Estimate (standard error)	Estimate (standard error)	Estimate (standard error)
Male gender	**0.3163 (.0653)**	**0.7991 (.1178)**	**0.4602 (.1203)**
Married	**0.3793 (.0640)**	**0.8491 (.1149)**	**0.7093 (.1246)**
Age < 45 years	−0.1401 (.0917)	**−30.8125 (.0080)**	**−0.6703 (.0262)**
Organizational Tenure < 15 years	0.2477 (954.48)	1.1063 (1773.45)	**0.2921 (.1184)**
Works Fulltime	**0.3827 (.0707)**	**0.8891 (.1230)**	**0.3757 (.1250)**
Works Part-time/Per Diem	**0.2852 (.0897)**	**1.0609 (.1861)**	**0.4195 (.1875)**
Doctor	0.0353 (.0718)	**0.4413 (.1315)**	0.1360 (.1302)
Advanced-Practice Clinician (APC)	**−0.1661 (.0629)**	**0.4915 (.1134)**	**−0.2498 (.1167)**
Works in Primary Care	**0.3484 (.0663)**	**1.0523 (.1299)**	0.0484 (.1444)
Not in Primary Care	**0.3331 (.0630)**	**0.8732 (.1141)**	**0.6652 (.1172)**
Workload ψ	**0.2335 (.0726)**	−0.1773 (.1464)	**−1.1792 (.1637)**
Autonomy Needs ψ	**−0.1559 (.0677)**	**0.2300 (.1107)**	**0.6356 (.1437)**
Relatedness Needs ψ	**−0.1528 (.0655)**	0.0593 (.1113)	**0.7209 (.1429)**
Meaningfulness of Practice ψ	0.0214 (.0626)	0.1381 (.0934)	**0.3828 (.1234)**
Risk Aversion ψ	−0.0453 (.0599)	**0.2256 (.1026)**	**−0.2603 (.1181)**
Intolerance of Uncertainty ψ	−0.0482 (.0659)	0.2384 (.1264)	**−0.4419 (.1238)**
Unit Support Staff F.T.E.s ψ	0.0156 (.0541)	0.0916 (.0999)	−0.1679 (.1176)
Clinical Unit of ≤ 5 Practitioners	**−1.6490 (.1106)**	**−26.1024 (.2091)**	−1.7937 (1.0024)

Note: Bolded figures indicate statistical significance at the alpha = 0.05 significance level

Ψ = This continuous variable was standardized

^a^These are fixed-effects-only zero-inflated beta regressions, on each independent variable, of the proportion of time that a provider was dissatisfied with their clinical practice

In the parsimonious mixed-effects one-inflated multivariable beta regression model of satisfaction frequency (Table 4a), the independently significant mean regressors were workload, relational needs, and meaningfulness; none of the covariates was an independently significant variance regressor; but significant, unadjusted associations with the zero-inflation were manifested by workload, meaningfulness, intolerance of uncertainty, and support staff FTEs on the clinical unit. The interpretation of the mean parameters is that, on average, a heavier workload was associated with decreased likelihood of being frequently satisfied, whereas higher gratification of autonomy and relatedness needs plus finding greater meaning in work were linked to increased frequency of satisfaction. The interpretation of results for the one-inflation submodel is that a heavier workload and greater intolerance for uncertainty are associated with decreased likelihood of being a highly satisfied practitioner, whereas finding greater meaning in patient care increases that likelihood.

**Table 4:** Multivariable inflated beta regression models

(a) Multivariable Mixed-Effects One-Inflated Beta Regression Model of Satisfaction with Practice^a^				
Sub-Model	Parameter	Estimate (standard error)	95 % Confidence Interval of Estimate	t statistic (*p* value)
Mean	Intercept (b_0_)	−0.1130 (.0609)	−0.2344, −0.0084	−1.85 (.0677)
	Workload ψ	−0.3400 (.0602)	−0.4600, −0.2200	−5.65 (<.0001)
	Autonomy needs ψ	0.1280 (.0721)	−0.0158, 0.2717	1.77 (.0802)
	Relatedness needs ψ	0.1898 (.0761)	0.0382, 0.3414	2.49 (.0149)
	Meaningfulness ψ	0.2372 (.0533)	0.1309, 0.3435	4.45 (<.0001)
Variance	Intercept (d_0_)	1.9907 (.0991)	1.7931, 2.1882	20.09 (<.0001)
One-Inflation	Intercept (one_0_)	−1.0837 (.1699)	−1.4233, −0.7452	−6.38 (<.0001)
	Workload ψ	−1.0294 (.1892)	−1.4065, −0.6523	−5.44 (<.0001)
	Meaningfulness ψ	0.6373 (.1750)	0.2885, 0.9861	3.64 (.0005)
	Intolerance of Uncertainty ψ	−0.4058 (.1600)	−0.7246, −0.0870	−2.54 (<.0001)
	Unit Support Staff F.T.E.s ψ	−0.3886 (.1832)	−0.7538, −0.0235	−2.12 (.0373)
(b) Multivariable Mixed-Effects Zero-Inflated Beta Regression Model of Dissatisfaction with Practice^b^				
Sub-Model	Parameter	Estimate (standard error)	95 % Confidence Interval of Estimate	t statistic (*p* value)
Mean	Intercept (b_0_)	−0.7590 (.0266)	−0.8119, −0.7061	−28.59 (<.0001)
	Workload ψ	0.2732 (.0601)	0.1534, 0.3929	4.55 (<.0001)
	Relatedness needs ψ	−0.2437 (.0592)	−0.3618, −0.1257	−4.12 (.0001)
Variance	Intercept (d_0_)	2.2825 (.1429)	1.9977, 2.5673	15.98 (<.0001)
	Autonomy needs ψ	0.3616 (.0961)	0.1701, 0.5531	3.76 (.0003)
	Risk Aversion ψ	0.2410 (.0941)	0.0535, 0.4285	2.56 (.0125)
Zero-Inflation	Intercept (zero_0_)	−1.1645 (.2906)	−1.7438, −0.5852	−4.01 (.0001)
	Not in Primary Care	0.6665 (.3464)	−0.0240, 1.3569	1.92 (.0583)
	Workload ψ	−1.1722 (.1932)	−1.5574, −0.7870	−6.07 (<.0001)
	Relatedness ψ	0.5691 (.1722)	0.2258, 0.9124	3.30 (.0015)
	Meaningfulness ψ	0.6738 (.1807)	0.3136, 1.0339	3.73 (.0004)
	Intolerance of Uncertainty ψ	−0.3895 (.1608)	−0.7101, −0.0689	−2.42 (.0179)

^a^ Goodness of Fit Statistics: -2 Log Likelihood = 113.3; AIC = 141.3; BIC = 173.6

^b^ Goodness of Fit Statistics: -2 Log Likelihood = 100.1; AIC = 130.1; BIC = 164.4

Ψ = This continuous variable was standardized

In the mixed-effects zero-inflated multivariable beta regression model of dissatisfaction frequency (Table 4b), the independently significant mean regressors were workload and relatedness; variance regressors that were independently significant were autonomy and risk aversion; and the significant zero-inflation regressors were workload, relatedness, meaningfulness, and intolerance of uncertainty. Working in a non-primary-care specialty had a marginally non-significant effect in the variance submodel. The inference from the mean submodel is that, on average, more fulfilled relatedness needs were associated with less frequent dissatisfaction. Higher workload, by contrast, was linked to more frequent dissatisfaction. The variance submodel implies that more fulfilled autonomy needs and greater risk aversion were associated with increased variation in the dissatisfaction frequency. The inference from the zero-inflation submodel is that greater fulfillment of relational needs, and a higher meaning in patient care increased the likelihood of being among the least frequently dissatisfied practitioners; a heavier workload and greater intolerance of uncertainty had the opposite effect.

## Discussion

We sought to investigate the factors that had the most significant predictive association with the proportion of time that group-employed, rural physicians and mid-level practitioners in upstate New York were satisfied or dissatisfied with practice. Practitioners with greater workloads and/or intolerance of uncertainty were less likely to enjoy top-quintile satisfaction; higher work meaningfulness increased that likelihood. Practitioners with more gratified relational needs and work meaningfulness were more likely to be in the bottom quintile of dissatisfaction. Heavier workloads and greater intolerance of uncertainty were linked to less likelihood of being in the bottom quintile in terms of dissatisfaction frequency.

The present study highlighted the role of workload as both a motivating and hygiene factor among clinicians. Subjective perceptions of workload quantity, rather than objective amounts of workload, drive the feelings of dissatisfaction [60, 82]. More intense workloads lead to heightened job stress or role strain, which diminishes professional satisfaction [83]. Thomassen and colleagues report that increased workload is linked to depression among rural physicians [84]. Lavanchy and colleagues cite manageable on-call shifts as one of the principal predictors of professional satisfaction [43]. The degree of autonomy permitted to the individual practitioner and the level of support rendered to them by their organization could moderate the effect(s) of workload on professional satisfaction and well-being [85].

Practitioners with more fulfilled relatedness needs were significantly more likely to enjoy frequent satisfaction, and to report being dissatisfied only a minimal amount (0–20 %) of the time. Relatedness was both a motivator and hygiene factor. There could be a selection effect whereby practitioners that elect to serve our rural communities were more motivated by the need to feel a strong sense of community and connectedness [34, 86]. Nevertheless, supportive professional relationships assist clinicians in coping with practice stress [87], thus reducing the likelihood of job distress and burnout [88]. The social support of their peers satisfies practitioners more than employee assistance programs initiated by managers [89]. Among employed practitioners, positive peer relationships influence quality of work life more powerfully than staff support, job control, income, or time pressure [90]. The quality of work relationships has also been linked to attrition from clinical practice [88, 91].

In this study, the extent to which practitioners perceived their autonomy needs as gratified had a positive association with the variance in dissatisfaction with practice. Autonomy was a hygiene factor rather than a motivator. Medical professionals require an optimum level of clinical autonomy in order to exercise the fiduciary duty of providing healthcare services with the interests of the patient being their foremost consideration [92]. Practitioners need strong autonomy support not just from their practice managers or supervisors, but also from their professional peers [93]. Studies consistently find significant associations between autonomy and job satisfaction among physicians [94–96] and advanced-practice clinicians [97–99]. The escalation of regulations and mandates during the current health reform era, coupled with the wave of institutional consolidations/amalgamations and trends towards individual practitioners choosing employment rather independent practice, has led to widespread erosion of individual autonomy [6]. In order to protect clinician well-being during systemic and institutional reform, clinicians and practice managers should collaborate to find innovative ways of sheltering professional autonomy [100]. Autonomy can be re-configured from an individualized to a group concept and exercised collectively or collaboratively [101, 102].

In our study, clinicians who derived greater intrinsic meaning from their practice had a significantly greater likelihood of being in the top quintile of satisfaction and/or being in the bottom quintile of dissatisfaction. Work meaningfulness was a motivator and a hygiene factor. Despite calls for enhancing meaning in clinical work as a means of improving professional wellbeing [103, 104], the construct of work meaningfulness has not been thoroughly investigated among medical practitioners. Theory and anecdotal evidence suggest that meaningful work is experienced as highly significant and holding positive intrinsic meaning for the practitioner [105]. In a narrative analysis of stories written by physicians about work-related experiences that they found meaningful, Horowitz and colleagues found 3 principal themes: making a connection with patients, impacting someone’s life, and a fundamental change in the doctor’s perspective [106]. Those who find high intrinsic meaning in their work often view their profession as a “calling” [107]. Cardador and colleagues found that physicians who saw medicine as a calling had greater commitment to their healthcare organization, since they viewed it as instrumental in helping them to achieve their deeply valued professional goals [108]. In another study, such physicians reported greater satisfaction with providing treatment for complex conditions such as smoking, alcoholism and obesity than their counterparts [109]. Among 220 certified nurse midwives, Brianna Caza found that high meaningfulness of the work was associated with reduced burnout [110]. Our study contributes to this growing empirical evidence of the underlying role of work meaningfulness in the quality of work life of medical professionals.

Practitioners that are highly intolerant of uncertainty/ambiguity were less likely to be in the top quintile of satisfaction and bottom quintile of dissatisfaction. Ambiguity tolerance functioned both as a motivator and hygiene factor. Our finding corroborates results from a study among Swiss physicians, in which uncertainty intolerance was associated with low job satisfaction [111]. Inability to tolerate ambiguity is implicated in fomenting job distress during medical training [112]. Among doctors, uncertainty intolerance is also associated with poor stewardship of healthcare resources [113], negative attitudes towards dying patients [114] or the underserved [115], poor diagnostic performance [116], and not adhering to medical evidence [117]. Those practitioners who tolerate uncertainty well find cognitively ambiguous clinical scenarios intellectually stimulating [118]. Since such uncertainties are commonplace in medical practice, those who see them as opportunities to grow can build their tolerance further [119, 120], enhancing the quality of their work life. Those who are intolerant of uncertainty seek to avoid ambiguous stimuli, which further degrades their ambiguity-processing capacity [119, 120], and undermines their professional satisfaction.

Risk aversion was significantly associated with variance in the dissatisfaction frequency but did not manifest significance in other sub-models. For purposes of brevity and due to space constraints, only two items of the risk-taking subscale were included in our survey, which may have limited the sensitivity of the measure. Risk tolerance refers to differential attention and reactions of individuals to stimuli in situations that are potentially risky. Risk-averse individuals differ from risk-takers in the way they evaluate a work setting [121], e.g. in their perceptions about the quality of work life. Risk-averse people put a high premium on procedural justice and fairness in an organization [122], which they see as restoring or maintaining predictability. Future studies should examine further the role that risk aversion plays in fomenting job stress among medical practitioners and how to avert or mitigate its effects.

Unlike the Great American Physician Survey [123], our study found no independent association between a practitioner’s age or employment tenure and their dis/satisfaction with practice. The significant associations of age and tenure with practice dis/satisfaction, in the univariate models, did not persist once we accounted for other covariates. Among the characteristics of a practice unit, only the level of support staffing manifested an independent effect. Serving on a unit with higher coverage by support staff was significantly associated with a reduced likelihood of being in the most satisfied quintile of practitioners. The level of support staffing on a unit could, however, reflect the clinical workload for which the unit is responsible. Differences in practice satisfaction by other demographic categories, such as gender, marital status, fulltime status, practice scope, and primary versus specialty/subspecialty practice, largely reflect confounding between individual demographics and the principal psychosocial factors described above.

### Strengths and limitations

Since we employed a survey methodology to collect data on various psychosocial and demographic factors, selection biases could limit the external validity of our findings. Our respondents did not systematically differ from non-responders on demographics, with the exception of median organizational tenure (respondents 7.8 years versus 4.8 for non-responders; *p* < 0.0001). The cross-sectional observation design of this study also means that we could not definitively establish the temporal evolution of dissatisfaction. The unique rural and small-town setting for the study is also a potential limit on external validity. Omitted variable bias is another potential downside, since we excluded some plausible constructs and limited the questionnaire’s length in order to prevent it from getting unwieldy, which can cause poor response rates. For non-physicians who have no independent practice, satisfaction with the supervising physician and acknowledgement of the PA/NP role by rural communities are two factors, highlighted by prior studies, which we did not measure. Our findings should be cautiously interpreted as limited, context-specific evidence until they are replicated in longitudinal studies and quasi experiments across multiple, diverse contexts or settings. Linking survey data to administrative information meant that our analysis included, not just self report variables but, objective measures too. An additional strength was the sophisticated analysis strategy that was robust to clustering in the sample (clinicians nested within practice units), the skewed (non-normal) distribution of the outcomes plus their configuration as proportions. Many similar studies make questionable assumptions that outcomes are linear or normally distributed (hence the frequent recourse to ordinary least squares regression), or use controversial methods of transforming the data to try and eliminate the skew. Our conservative approach rested on as few unsupported assumptions as possible. The response rate (65.1 %) to our survey compares favorably with those reported by other studies of medical professionals, a further strength.

#### Implications

This study emphasizes the importance of continuous tracking of practitioner satisfaction (which is as important as patient satisfaction, a metric already being tracked regularly) by practice leaders and healthcare organization managers. The proportion of time that practitioners, were satisfied or dissatisfied had a predictive association with mutable factors such as workload, autonomy and relational needs, work meaningfulness, attitudes towards uncertainty/ambiguity and risk taking or aversion, and the support staffing. Healthcare organizations should redesign systems to enhance inter-professional, multidisciplinary teamwork as a strategy for alleviating perceptions of inequity in the division of clinical labor and fulfilling the practitioners’ relational needs. Managers of health institutions should help to build supportive peer networks or professional communities that increase social capital and reduce loneliness/isolation, particularly among practitioners in rural settings. Practitioner groups or associations should negotiate with healthcare organizations and third party payers in order to find innovative ways of balancing professional autonomy with accountability. Leadership coaching should be given to practitioners to enhance their skills of dealing with situations with uncertain and potentially risky outcomes. Training in ambiguity tolerance skills ought to be incorporated within medical education curriculums [124]. Awareness campaigns can use social marketing strategies to “market” a professionalism built on a healthier balance between work life and personal/family life among health practitioners.

## Conclusion

Group-employed, rural practitioners who report having a higher workload and being intolerant of clinical ambiguity are less likely to belong to the most frequently satisfied and least frequently dissatisfied quintiles, but finding patient-care inherently meaningful and having more of one’s relational needs fulfilled increases that likelihood. Workload, relatedness, meaningfulness and ambiguity intolerance are both motivators and hygiene factors. Support staffing is a motivator, while autonomy and risk aversion are hygiene factors. Once these mutable factors are accounted for, individual practitioner demographics and most practice unit characteristics do not manifest an independent association with the frequency of dis/satisfaction with rural practice.

## Abbreviations

ACOs: Accountable-Care Organizations
AIC: Akaike Information Criterion
APC: Advanced-practice clinician
BIC: Bayesian Information Criterion
CI: Confidence interval
CNAs: Certified nurse assistants
FTEs: Full-time equivalents
GAMLSS: Generalized additive model for location, scale and shape
IRB: Institutional Review Board
LPNs: Licensed practical nurses
NC: North Carolina
NLMIXED: Non-linear mixed regression procedure
NP: Nurse practitioner
PA: Physician assistant
PCAs: Personal care assistants
PRAWS: Practitioner Resilience, Adaptability and Well-being Study
Q_1_ or q1: first quartile
Q_3_ or q3: third quartile
RNs: Registered nurses
SAS™: Statistical Analysis Software
SDT: Self-determination theory
U.S.: United States

## Footnotes

1 The one-inflation submodel assessed the probability that the frequency of satisfaction reached or exceeded 80 %; whereas the zero-inflation submodel assessed the probability that frequency of dissatisfaction did not exceed 20 %.
